# The effect of pre–measurement rest time on systolic ankle pressure measurements

**DOI:** 10.1186/1757-1146-6-S1-P2

**Published:** 2013-05-31

**Authors:** Vivienne Chuter, Sarah Casey

**Affiliations:** 1 Podiatry Discipline, University of Newcastle, Ourimbah, NSW, 2258, Australia

## Background

Systolic ankle pressures are measured as part of an ankle-brachial index (ABI) to screen for the presence of peripheral arterial disease (PAD). Despite widespread use of the ABI, there is currently no research evidence investigating the amount of pre-measurement rest required for the systolic ankle pressure to stabilise.

## Methods

One hundred and forty participants meeting current guidelines for screening for PAD volunteered for this study. Following 5 minutes of rest in the supine horizontal position, ankle systolic pressures of the left or right lower extremity were taken using hand-held Doppler. Measurements were repeated at 10 and 15 minutes. Testing was repeated 7-10 days later.

## Results

A significant drop in ankle pressure of 5.02 mmHg occurred between 5 and 10 minutes (p<0.05) however no significant change occurred between 10 and 15 minutes (mean change 0.15 mmHg, p=0.99). Presence of diabetes was associated with a smaller drop between 5 and 15 minutes (mean change 1.85 mmHg) and predicted 14% of the variance in change in ankle pressure (beta=-3.72, p>0.05). Test-retest reliability after 5 minutes was excellent (intraclass correlation co-efficient (ICC): 0.84) however increased for measurements taken at 10 and 15 minutes (ICC: 0.89 and 0.89 respectively).

## Conclusions

Results suggest ankle systolic pressures stabilise after 10 minutes of rest. Longer periods of pre-measurement rest did not improve reliability of the measurement significantly. Presence of diabetes affects ankle pressure changes in response to rest, however further investigation is required to identify the cause.

